# Standard for the Quantification of a Sterilization Effect Using an Artificial Intelligence Disinfection Robot

**DOI:** 10.3390/s21237776

**Published:** 2021-11-23

**Authors:** Heeju Hong, Wonkook Shin, Jieun Oh, Sunwoo Lee, Taeyoung Kim, Woosub Lee, Jongsuk Choi, Seungbeum Suh, Kanggeon Kim

**Affiliations:** Artificial Intelligence and Robotics Institute, Korea Institute of Science and Technology, Seoul 02792, Korea; hhj3553@gmail.com (H.H.); goddnjsrnr@kist.re.kr (W.S.); jieunoh@postech.ac.kr (J.O.); sunwoolee0@yonsei.ac.kr (S.L.); tyoung960302@gmail.com (T.K.); fieldrobot@gmail.com (W.L.); cjs@kist.re.kr (J.C.)

**Keywords:** COVID-19, deep learning, disinfection robot, object detection, ultraviolet disinfection (UVD)

## Abstract

Recent outbreaks and the worldwide spread of COVID-19 have challenged mankind with unprecedented difficulties. The introduction of autonomous disinfection robots appears to be indispensable as consistent sterilization is in desperate demand under limited manpower. In this study, we developed an autonomous navigation robot capable of recognizing objects and locations with a high probability of contamination and capable of providing quantified sterilization effects. In order to quantify the 99.9% sterilization effect of various bacterial strains, as representative contaminants with robots operated under different modules, the operating parameters of the moving speed, distance between the sample and the robot, and the radiation angle were determined. We anticipate that the sterilization effect data we obtained with our disinfection robot, to the best of our knowledge, for the first time, will serve as a type of stepping stone, leading to practical applications at various sites requiring disinfection.

## 1. Introduction

The novel COVID-19 virus has led to highly contagious viral infections and is threatening lives across the world. According to the World Health Organization (WHO), there are approximately 166 million confirmed cases overall along with 3.44 million deaths reported as of 20 May, 2021 [1]. Due to the high contagiousness and mortality rate of this viral infection, disinfection strategies are strongly required. When a bacterial co-infection occurs with COVID-19, which is caused by severe acute respiratory syndrome coronavirus 2 (SARS-CoV-2), as the disease progresses, patients typically require intensive care and treatment with antibiotics. Moreover, in such cases, the mortality risk increases by 2.5 times due to uncontrolled sepsis, leading to the patient’s death [2]. This demonstrates the possibility of compound effects of SARS-CoV-2 with a bacterial infection; thus, establishing substantial requirements for disinfection strategies for both viruses and bacteria.

Infectious diseases are likely to be transmitted by direct or indirect contact with contaminants [3]. SARS-CoV-2 at 20 °C is reported to remain viable for 28 days on common surfaces under controlled experimental conditions [4,5], which makes the disinfection of viruses and bacteria on contaminated surfaces a crucial part of sterilization. Among various methods of surface disinfection, viral inactivation by UV-C irradiation is one of the most effective methods. UV-C can effectively eradicate pathogens and has advantages over other disinfection methods, given its shorter operating time and higher adaptability, allowing it to be used with automated electronic devices without any harmful effects on the system.

In order to enhance the disinfection efficiency, a consistent and differential space sterilization strategy based on the contamination probability is desired. However, achieving this goal by means of conventional quarantine would be difficult due to manpower limitations. To address this issue, we introduce an autonomous disinfection robot equipped with UV-C. Image processing technology also enables the system to recognize high-risk contaminated spaces and, thus, apply differential sterilization strategies. The standards for an operating ultraviolet disinfection (UVD) robot, which satisfies the complete sterilization state (99.9% sterilization effect), should be established through carefully designed experiments with actual contamination. It would be highly beneficial for users who require established standards to operate another UVD robot with different specifications and do not have access to the actual contamination experiments.

Robots have long served industrial applications, ranging from car manufacturing to package sorting. Robotic applications are gradually moving closer to the end user. The severity of the pandemic has proved that healthcare workers require all possible help, whether from humans or robots. Research related to robotics around the world is stepping up with regard to the development of robots that can help prevent the spread of COVID-19 [6,7]. There are various types of robots, such as drones [8,9], humanoids, legged robots [10], and mobile-based robots [11], all of which have different characteristics. Drones patrol areas and enforce lockdowns, assisting police officers [12]. Humanoid robots assist hospital staff by delivering medicine to patients, for instance, in the form of legged robots to take care of patients remotely. Mobile robots are used to disinfect public areas, such as airports and hospitals [13]. In such places, mobile robots do not have to move on all-terrain, as with legged robots, and they have great stability and movement efficiency. In particular, autonomous mobile robots are capable of autonomous navigation without external guidance. Mobile robots equipped with autonomous driving functions are commonly utilized to perform disinfection tasks. For example, hospitals, offices, and public spaces must halt the spread of the virus and maintain a clean and healthy environment. For such purposes, UVD robots can be utilized to help maintain a clean environment [14,15,16].

In this study, we develop a UV-C equipped mobile robot system that disinfects viruses and bacteria on surfaces and validate its sterilization efficacy by quantifying its microbicidal performance using the bacterial strains of *Staphylococcus aureus* (*S. aureus*), *Pseudomonas aeruginosa* (*P. aeruginosa*), and *Escherichia coli* Nissle 1917 (*E. coli*). The disinfection robot was operated until the system achieved a sterilization rate of 99.9% (3-log reduction), which is the standard of quarantine authorities. The operating parameters of the mobile robot used here were accurately quantified because the established disinfection data would serve as a type of basic parameter setting criteria for another UVD robot to be deployed in the field for future practical applications.

## 2. Materials and Methods

### 2.1. Robot System

**Figure 1:**
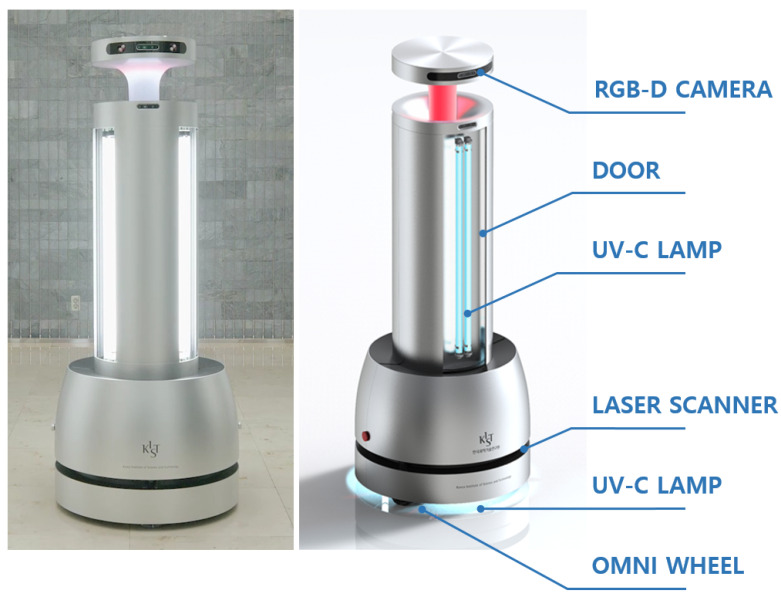
AIDBOT configuration.

Artificial Intelligence Disinfection roBOT (AIDBOT), a UVD robot with five RGB-D cameras, three laser sensors, and UV-C lights, is shown in Figure 1. The RGB-D cameras mounted on the robot are the Intel RealSense depth camera D435, which can obtain the depth information corresponding to the color image, and the resolution is 1280×720. In addition, the 2D LiDAR sensors SICK TiM551 are used for obstacle avoidance and driving, with an angular resolution of 1${}^{\circ }$ and a scanning frequency of 15 Hz. AIDBOT is an autonomous disinfection robot that can essentially drive autonomously using multiple cameras and laser sensors and can sterilize objects with UV-C light. In addition, AIDBOT uses a system capable of implementing sterilization strategies while considering the contact frequency. The system is mainly composed of three modules that handle autonomous driving, object detection, and path planning.

The autonomous driving module utilizes the Simultaneous Localization and Mapping (SLAM) [17,18] method. SLAM has received increased attention owing to its wide range of applications, e.g., AR/VR and autonomous driving robots. Visual SLAM is a SLAM method that uses camera sensors, and various visual SLAM methods have been researched recently [19]. AIDBOT applies TIMA-SLAM [20], a visual SLAM approach that uses multiple RGB-D cameras.

Using this SLAM method, the proposed AIDBOT obtains a map of the surrounding area and estimates the current robot’s location at the same time. The map and current robot’s location are used in the path planning module. This module also controls the robot, allowing it autonomously to drive along the path calculated by the path planning module.

The object detection module is in charge of detecting objects. Objects detected by AIDBOT are those that must be disinfected. In the field of object detection, researchers using deep learning have shown good performance outcomes. It is crucial for object detection deep learning models of disinfection robots to increase accuracy and work in real-time at the same time. Among the numerous object detection models currently available, we used the YOLOv4 [21] model to find target objects. The YOLOv4 model has improved accuracy with various deep learning techniques, such as Bag of Freebies and Bag of Specials. It also runs in real-time on the robot’s embedded system. Considering the frequency of contact, we selected various objects (e.g., chair, desk, button, handle), including person, and trained the YOLOv4 model to detect these objects. RGB-D cameras can capture RGB images with a depth map, which contains a distance value for each pixel. Using these properties, we calculated the location of the detected object. By adding the detected object’s location to the map, we were able to obtain a map reflecting the location information of the object to be disinfected.

The path planning module calculates the optimal path based on the map and the robot’s current location as obtained from the autonomous driving and object detection modules. The optimal path is the route by which to disinfect the entire area, guaranteeing a 99.9% sterilization effect. To ensure a 99.9% sterilization effect, the path must be planned so as to reflect various disinfection strategies. For example, intensive disinfection strategies can be used by slowing down the robot’s speed in spaces where disinfection targets are concentrated. AIDBOT can operate at controlled speeds between 0.05 and 0.3 m/s. A path including various disinfection strategies is calculated based on a map reflecting the disinfection targets and considering proper speed control.

We used the dynamic window approach [22], one of the most popular algorithms for obstacle avoidance. It is a real-time obstacle avoidance algorithm that selects the angular and linear velocity pairs that can be quickly driven to a target point while avoiding obstacles.

**Figure 2:**
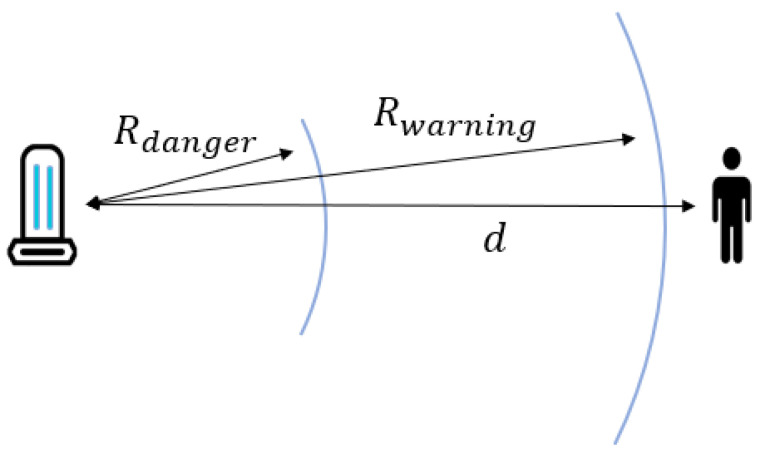
The dangerous radius of $R_{danger}$ and the warning radius of $R_{warning}$ of the UVD robot are set. The UVD robot detects a human and measures the distance of *d*.

AIDBOT utilizes the above three modules to perform autonomous disinfection works. In particular, a safety system is required because it sterilizes using UV-C lights that are very harmful to the human body. AIDBOT has a safety system that detects people within a certain range, and warns them or turns off the UV-D lights (Figure 2). Even with this safety system, it is not efficient to operate AIDBOT in crowded environments. So, AIDBOT operates at night when there are few people, or after work, minimizing the impact of crowded environmental constraints and enabling efficient disinfection work.

### 2.2. Target Object Recognition

We selected the objects that the robot needed to detect, in this case, a chair, a desk, a button, a handle, and a human. Chairs and desks, buttons, and handles have a high probability of germs due to frequent contact with human hands. In addition, UV-C is harmful to the human body, so the UVD robot must detect humans to stop disinfecting if humans are nearby [23]. When the UVD robot detects a human within a warning radius of 10 m, the UVD robot stops moving and alerts people through a warning sound. In particular, if the UVD robot detects a human within a dangerous radius of 5 m, the UVD robot turns off the UV-C lamp and closes the door (Figure 2). We collected images that include these target objects. We used the SUN RGB-D dataset [24], an indoor dataset, and data that we collected ourselves to fit our purpose. We used 7132 images for training and 773 images for testing. This deep learning model, trained for our purpose, was used to find the target object’s location in the UVD robot camera. Because we used a depth camera, we immediately determined the distance between the target object and the UVD robot.

### 2.3. Bacteria Culture

Three different bacterial strains were used for the sterilization effect experiments. These were *Staphylococcus aureus*, *Escherichia coli* Nissle 1917, and *Pseudomonas aeruginosa*, (kindly provided by Jung-Hyun Lee at Korea University). A small fragment of bacteria was inoculated in 10 mL of Luria-Bertani broth (LB, 1% *w*/*v* of tryptone, 0.5% *w*/*v* of sodium chloride, and 0.5% *w*/*v* of yeast extract, adjusted to pH 7.0). The bacterial cultures were incubated at 37 °C and 100 rpm in a shaking incubator overnight. The overnight cultures were diluted at 1% *v*/*v* and grown in fresh LB at 37 °C and 100 rpm in the shaking incubator. Once the bacteria were confirmed to be in the log phase ($OD_{600}$ at 1.2), they were set to $OD_{600}$ 1.0, which contains $1.16\times 10^{8}$ cfu/ml of *S. aureus*, $9.90\times 10^{6}$ cfu/mL of *E. coli*, and $2.01\times 10^{8}$ cfu/mL of *P. aeruginosa* prior to the experiments.

### 2.4. Microbicidal Assay Using UVD Robot

**Figure 3:**
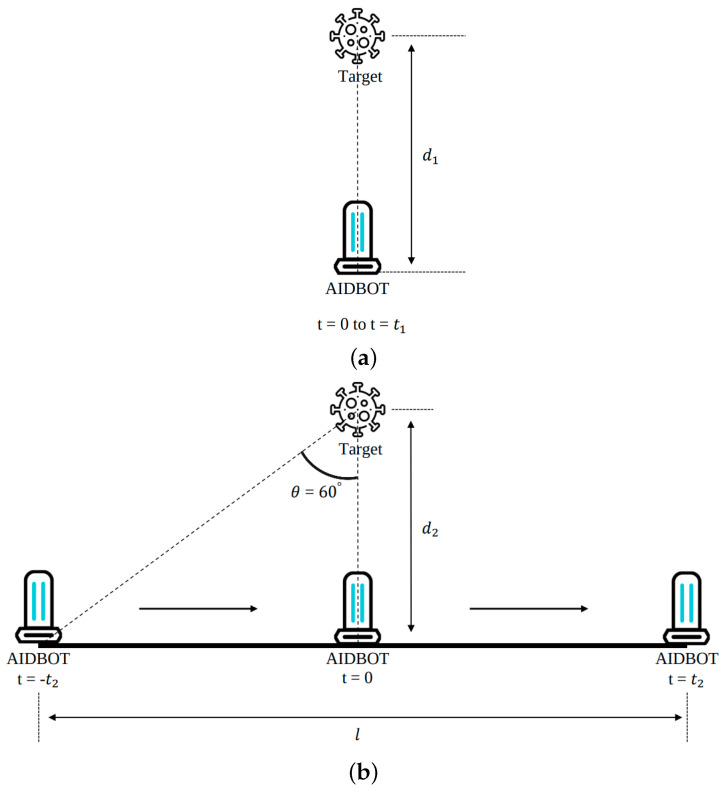
Illustration of two disinfection experiments with the UVD robot (**a**) Without moving, the UVD robot disinfects a target $d_{1}$ meters away for $t_{1}$ seconds. (**b**) At a velocity of $v_{2}$, the UVD robot disinfects a target $d_{2}$ meters away for $t_{2}$ seconds ($d_{2}$: vertical distance between the target and the UVD robot’s path).

After undertaking serial dilution of the bacterial cultures, $4.63\times 10^{3}$ cfu of *S. aureus*, $3.96\times 10^{3}$ cfu of *E. coli*, and $4.03\times 10^{3}$ cfu of *P. aeruginosa* were spread on separate agar plates using sterile spreader. Once bacterial samples on the agar plates for each stain were prepared, the distance between the plates and the UVD robot were fixed and two experiments were conducted depending on the two parameters of the UV-C radiation time (s) and the moving velocity of the UVD robot (m/s) (Figure 3).

For the first experiment, the plates were radiated with UV-C for 4, 8, 12, and 16 s in order to quantify the sterilization effect based on the irradiation duration. All samples were processed in triplicate and were positioned 1 m away from the UV-C source. After the UV-C light irradiation step was completed, the plates were incubated in 37 °C incubators overnight. The surviving bacterial colonies on the plates were enumerated and compared with un-irradiated cases to determine the microbicidal effect.

Once the sterilization effect of UV-C with the stationary UVD robot was evaluated (Figure 3a), the microbicidal effects of the UVD robot when moving at various speeds were evaluated in subsequent experiments (Figure 3b). The speed of the UVD robot was determined through the calculated travel distance of the robot considering the UV-C radiation angle and the equivalent UV-C radiation duration (energy) that sterilizes each bacterial strain to a rate of 99.9% (3-log reduction) (Equations (1) and (2)). The determined speed of the UVD robot that resulted in 3-log reduction disinfection for *E. coli* was 0.13 m/s. In order to observe the overall range of the sterilization effects, we also conducted tests at higher and lower speeds by a UVD robot as compared to the calculated speed. With this computed speed, we can also calculate the time it takes to disinfect a certain area.

(1) $$Energy\propto \frac{1}{distance}$$

(2) $$\frac{E}{d_{1}}\times t_{1}=\int_{-t_{2}}^{t_{2}}\frac{E}{\sqrt{d_{2}^{2}+{\left(v_{2}t\right)}^{2}}}dt$$

where $d_{1}$ is the distance in meters between the plates and the UVD robot when the robot is not moving; $t_{1}$ is the UV-C radiation time in seconds when the robot is not moving; $d_{2}$ is the vertical distance in meters between the plates and the robot when the robot is moving; $t_{2}$ is half of the total robot moving time in seconds; $v_{2}$ is the velocity, in meters per second, of the UVD robot; and E is the UV-C light irradiance in watts per square meter. As shown in Equation (1), energy and distance are inversely proportional. In Equation (2), since the distance between the plates and the robot changes when the robot moves, the corresponding energy change is integrated and compared with when the robot is stopped.

## 3. Result

### 3.1. Recognition of Intensive Bacterial Distribution Targets

**Figure 4:**
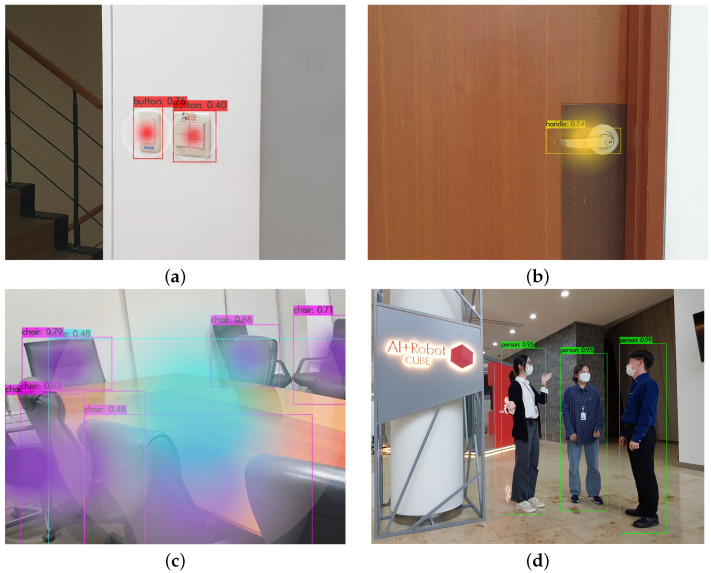
Target object detection results: (**a**) button, (**b**) handle, (**c**) chair and table, (**d**) person. The color space in the bounding box visualizes the bacterial distribution on the target object.

Our goal was the recognition of four objects that required intensive disinfection and one object that should not be disinfected. We trained the YOLOv4 model to detect these objects. To visualize the results, we decided to draw a bounding box for each object predicted by the deep learning model and to display a confidence score. Confidence scores indicate how confidently an object is predicted when a deep learning model makes a prediction. Usually, confidence scores are between 0 and 1, and the higher the score, the greater the confidence of the prediction by the deep learning model. We set the threshold of the confidence score to 0.25 in our object detection model. When the confidence score predicted by the object detection model exceeds the threshold, we display a bounding box and the confidence score for each object. As shown in Figure 4, our model detected disinfection targets and displayed a bounding box and confidence score. The model recognized two buttons with confidence scores of 0.76 and 0.40 and properly displayed the bounding box and confidence score, respectively (Figure 4a). We compared it with EfficientDet-D3 [25], which is known for showing good accuracy and enough speed to work in real-time. As a result, we confirmed that YOLOv4’s mAP was slightly higher than EfficientDet-D3 when trained with our own dataset. Moreover, we tested our YOLOv4 model with 150 images and confirmed that the accuracy of button, handle, desk, chair, and person was 90%, 93.3%, 90%, 93.3%, and 100%, respectively.

### 3.2. UV-C Sterilization Effect

The sterilization assay using the UVD robot shows that more effective sterilization occurred when either longer UV-C exposure times were applied at a fixed distance or a slower moving speed of the UVD robot operation was used, as expected. Regarding the quantitative disinfection effect, the UV-C light needed to be applied for approximately 11 s for *S. aureus*, 15 s for *P. aeruginosa*, and 19 s for *E. coli* to achieve a 3-log reduction (99.9% sterilization effect) at a distance of 1 m. Although there were slight differences in the UV-C sterilization effects on each strain, the targeted sterilization effect could be attained within 20 s of UV-C application. The corresponding UV-C dose ranges from 15 to 28 J/m${}^{2}$. It is important to note that the accumulative UV-C energy used for each sample is logarithmically proportional to the number of surviving bacteria over the initial population. The survival rate can therefore be estimated through the determined linear relationship according to the UV-C energy dose used for each strain.

**Figure 5:**
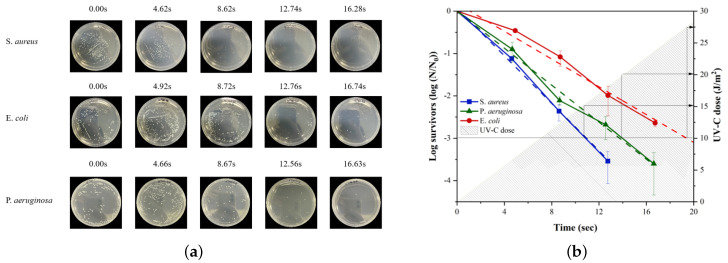
Stationary UV-C (254 nm) sterilization experiment with a prolonged exposure duration at a 1 m distance: (**a**) bacterial plates and (**b**) sterilization effect (log survivors) graph where the duration of eh UV-C application varies.

**Figure 6:**
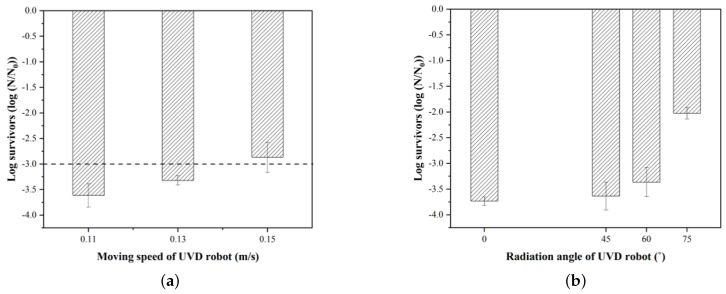
UV-C sterilization effect (log survivor) results to determine the 3-log reduction of bacteria; (**a**) varying the moving speed of the UVD robot; (**b**) the radiation angle of the UVD robot.

The sterilization experiments with the moving robot for *E. coli*, which required the highest level of UV-C energy or sterilization in a previous experiment (Figure 5b), showed that the 3-log reduction disinfection was achieved (Figure 6) when the robot moves at the calculated moving speed of 0.13 m/s. Additional moving speeds (0.11 m/s and 0.15 m/s) also showed expected disinfection results (Figure 6a). The disinfection effect was slightly higher than the expected rate because the radiation angle of the UV-C light exceeded the expected angle of 60 degrees (Figure 6b), causing more UV-C energy to be exposed to the sample. A sterilization assay considering the UVD robot movement shows that the robot operating at a speed of 0.13 m/s or lower, 1.0 m away from the wall guarantees a 3-log reduction sterilization effect. Additionally, the results show that decreasing the robot’s moving speed by 0.01 m/s yields a greater disinfection effect by approximately 1.2 times.

**Figure 7:**
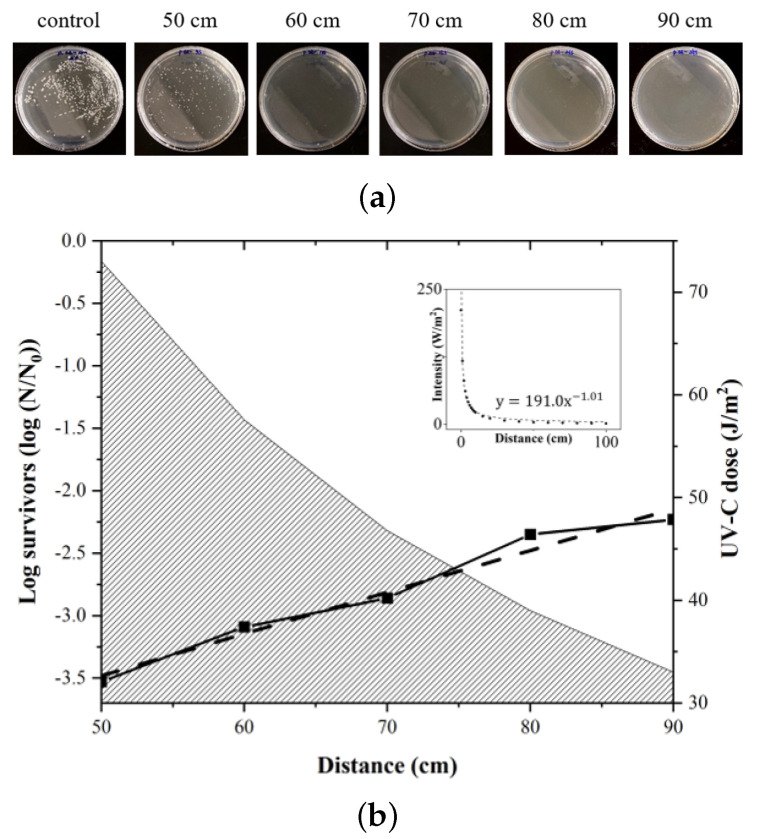
In order to demonstrate the linear relationship between the UV-C dose and the disinfection effect (log survivors of bacteria), which is inversely proportional to the distance between the UV-C source and the sample, the sterilization effects of *P. aeruginosa* at varying distances (50, 60, 70, 80, and 90 cm) were estimated with the equivalent disinfection experiment, described as Section 2.4 with stationary UV-C light condition. (**a**) Bacterial plates; (**b**) disinfection effect (log survivors) where the distance between the UV-C light and the bacteria plates varies with the corresponding UV-C dose are shown. The inset figure shows the inverse relationship between the distance and the UV-C intensity.

Given that the accumulative UV-C energy exposed onto the sample is logarithmically proportional to the number of surviving bacteria over the initial population shown in the UVD robot sterilization experiment (Figure 5), the logarithmic value of the fraction of surviving bacteria is directly proportional to the exposure duration due to the directly proportional accumulative UV-C dose (J/m${}^{2}$). Regarding the distance between the UV-C source and the sample, the UV-C energy dose is inversely proportional to the distance and the logarithmic value of the fraction of the surviving bacteria therefore sustains this relationship as well (Figure 7b). Therefore, it is vital to understand the trade-off between the operation of the UVD robot and the adaptive application of the optimized operating strategies according to certain environmental features, including the presence of obstacles, human traffic, the sterilization frequency, and the contaminant distribution, among others.

### 3.3. Robot Simulation

**Figure 8:**
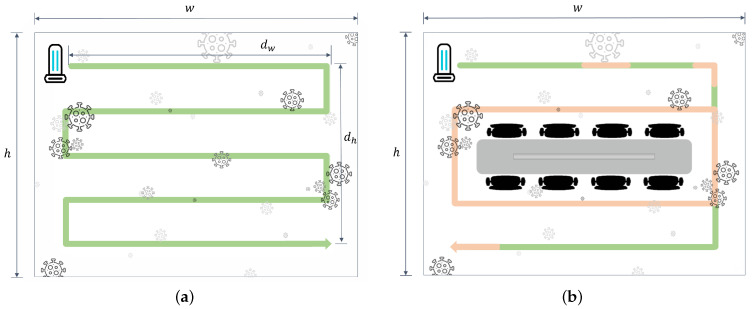
Illustration of a virtual conference room with a width and height of *w* and *h*, respectively. In the case of (**a**), an empty conference room and (**b**) a general conference room, the expected paths of the UVD robot are correspondingly indicated by the green and pink lines. The green lines denote the path moving at a speed of 0.13 m/s, and the pink lines are the path requiring intensive sterilization.

Given that UVD robot operation at a rate of 0.13 m/s or lower, 1.0 m away from the target guarantees the 3-log reduction sterilization effect, a sterilization simulation of a virtual conference room was carried out (Figure 8).

For quantitative verification, we assumed an empty conference room *w* of 12 m by *h* of 10 m in size and a UVD robot operating at a speed of 0.13 m/s (Figure 8a). Based on our verification, the path for disinfecting the entire conference room is expressed as the green line. Calculating the path that prevents overlapping of a 1 m distance results in a size of $d_{w}$ of 10 m by $d_{h}$ of 8 m. The total path is calculated as 58 m, and considering the robot operating at a speed of 0.13 m/s, the process takes seven minutes and 45 s.

However, in an actual environment, various objects, such as chairs and tables, exist in such a conference room. In this case, the path is expected to be simplified and shortened (Figure 8b). However, there may be places where objects to be disinfected are concentrated. Under these conditions, the robot’s speed must be reduced to ensure the 3-log reduction sterilization effect. Through experiments, we verified that decreasing the robot’s moving speed by 0.01 m/s yields approximately a 1.2 times greater disinfection effect (Figure 6a). The pink lines indicate a path that requires intensive disinfection, for which it is assumed that the UVD robot moves at a speed of 0.10 m/s and guarantees a 3-log reduction sterilization effect, where contaminants within the concentrated infected area may exceed five times those in a regular area [26,27]. The total path is calculated as 52 m (18 m for the green line and 34 m for the pink line), and considering the UVD robot operating at a speed of 0.13 m/s along the green line and at 0.10 m/s along the pink line, the process takes approximately eight minutes. Compared to the case of an empty conference room, it was confirmed that the total path decreased, whereas the total disinfection time increased. In conclusion, through the two aforementioned assumptions, we confirmed that it takes approximately eight minutes to disinfect a 120 m${}^{2}$ area, while ensuring a 3-log reduction sterilization effect. However, the total disinfection time can vary depending on the path and the distribution of the disinfection target.

## 4. Discussion and Conclusions

Traditional methods of sterilizing bacteria and viruses using UV-C rely on fixed or human-transported UV-C sterilizers. For example, there are UV-C sterilizers for disinfecting tableware, as well as portable sterilizers, such as toothbrush cases. These conventional portable UV-C sterilizers, however, are not suitable for disinfecting large areas where various viruses and bacteria exist as contaminants, especially when humans are nearby, as UV-C light exposure can be harmful to humans. Thus, sterilization operations with UVD robots in large spaces would be a much safer and more effective strategy. Deploying autonomous UVD robots equipped with computer vision and object recognition through an image processing algorithm can help to sterilize an area more efficiently; UVD robots can detect objects, which are highly likely to be contaminated, such as handles and buttons, and can disinfect these objects intensively under longer exposures times of the UV-C light. Therefore, utilizing autonomous UVD robots can enable consistent and safe quarantine routines and, hence, can improve public health and alleviate the problem of medical personnel shortages.

When using robots at actual quarantine sites, longer sterilization times are required for objects with high levels of contamination by viruses or bacteria. From our experiment, the appropriate robot speed and UV-C light exposure time could be calculated. According to the object detection result, the UVD robot’s speed and exposure time of the UV-C light should be adjusted in order to sterilize targets that are highly contaminated by viruses and bacteria. When the UVD robot passes by targets with high bacterial distributions, the robot recognizes such a situation and should slow down or move closer to the target for intensive sterilization.

Although the UV-C dose for 99.9% disinfection of COVID-19 is not specifically reported, the UV-C dose for inactivating 99.9% of comparable SARS family viruses is known to be 100–200 J/m${}^{2}$ under controlled laboratory conditions [28]. This is approximately 3.6 to 7.1-fold of the dose required to disinfect 99.9% of *E. coli*. Disinfecting a large area with the developed UVD robot in the case of a certain contaminant could be precisely implemented by adjusting the moving speed because the disinfection effect of the UVD robot operation was accurately quantified in this study.

We have established the UVD robot operational standards with carefully designed disinfection experiments to resolve the challenges of UVD robot users who do not have access to experimentally quantified disinfecting effects. Therefore, the established disinfection data would serve as a type of basic parameter setting criteria for another UVD robot to be deployed in the field for future practical applications.

There are a couple of considerations when operating UVD robots in the field. First, the robot operating time and frequency should be carefully selected due to both the harmful effects of UV-C light (254 nm) on humans nearby and due to the possible repopulation of remaining bacteria or newly introduced contamination occurring again at sites after sterilization. Recently developed far UV-C (222 nm) has a less harmful effect but lower sterilization effect; it could be selectively used for different operation times when there are humans nearby [29]. The humidity and surface features should also be considered because the UV-C sterilization effect can be altered. For example, most Gram-negative bacteria are especially sensitive to the relative humidity as they have a relatively thin peptidoglycan layer (5–10 nm thick) compared to Gram-positive bacteria (30–100 nm thick) and are thus more likely to undergo dehydration and desiccation [30,31]. It has also been reported that UV-C radiation under the soil, in dust, and in bodily fluids can also interfere with disinfection [32]. The prevalent methods of eradicating bacterial/viral infectious agents include not only UV-C, but also chemicals. More effective sterilization can be ensured when chemical disinfectants are also used in the quarantine procedure. Specifically, in an environment where UV-C sterilization is not sufficiently nor evenly transmitted due to various types of obstructing structures located in the area, utilizing the auxiliary quarantine strategy of chemical disinfectants would be useful. Accordingly, various modes of sterilization should be deployed with optimized path planning strategies considering the relevant spatial information in future studies. Examples of such variables include the size of the space, a probability distribution map of the infecting agents, and obstructing structure locations.

## Data Availability

The data presented in this study are available upon request.
